# Translational Control by the DEAD Box RNA Helicase *belle* Regulates Ecdysone-Triggered Transcriptional Cascades

**DOI:** 10.1371/journal.pgen.1003085

**Published:** 2012-11-29

**Authors:** Robert J. Ihry, Anne L. Sapiro, Arash Bashirullah

**Affiliations:** 1 Division of Pharmaceutical Sciences, University of Wisconsin–Madison, Madison, Wisconsin, United States of America; 2 Cellular and Molecular Biology Graduate Program, University of Wisconsin–Madison, Madison, Wisconsin, United States of America; University of California San Francisco, United States of America

## Abstract

Steroid hormones act, through their respective nuclear receptors, to regulate target gene expression. Despite their critical role in development, physiology, and disease, however, it is still unclear how these systemic cues are refined into tissue-specific responses. We identified a mutation in the evolutionarily conserved DEAD box RNA helicase *belle/DDX3* that disrupts a subset of responses to the steroid hormone ecdysone during *Drosophila melanogaster* metamorphosis. We demonstrate that *belle* directly regulates translation of *E74A*, an ets transcription factor and critical component of the ecdysone-induced transcriptional cascade. Although *E74A* mRNA accumulates to abnormally high levels in *belle* mutant tissues, no E74A protein is detectable, resulting in misregulation of E74A-dependent ecdysone response genes. The accumulation of *E74A* mRNA in *belle* mutant salivary glands is a result of auto-regulation, fulfilling a prediction made by Ashburner nearly 40 years ago. In this model, Ashburner postulates that, in addition to regulating secondary response genes, protein products of primary response genes like *E74A* also inhibit their own ecdysone-induced transcription. Moreover, although ecdysone-triggered transcription of *E74A* appears to be ubiquitous during metamorphosis, *belle*-dependent translation of *E74A* mRNA is spatially restricted. These results demonstrate that translational control plays a critical, and previously unknown, role in refining transcriptional responses to the steroid hormone ecdysone.

## Author Summary

Pulses of steroid hormones regulate a variety of biological processes, but how these simple global cues are converted into specific local responses remains unclear. While steroid responses have traditionally been thought to be regulated at the transcriptional level, here we demonstrate that translational control plays a novel role in refining steroid signals. The DEAD box RNA helicase *belle* directly regulates the translation of *E74A* mRNA, which encodes a transcription factor that is induced by the fly steroid hormone ecdysone and then rapidly repressed. This process is disrupted in *belle* mutant tissues, where *E74A* mRNA accumulates to abnormally high levels but is not translated. We demonstrate that Belle-dependent translation of *E74A* is required to both repress its own transcription and to induce tissue-specific target genes. These findings confirm the prediction that auto-regulation is important for the self-limiting behavior of steroid responses and demonstrate a critical role for translational control in refining a global hormonal signal into specific biological responses.

## Introduction

Steroid hormones regulate a wide variety of biological responses during development and physiological homeostasis. These small lipophilic compounds act, through their respective nuclear receptors, to directly regulate target gene transcription. This initial transcriptional response occurs within minutes and does not require new protein synthesis; within hours of hormone exposure, however, expression of a larger set of genes is initiated, this response does require new protein synthesis. This hierarchical model of steroid-induced transcriptional responses was originally proposed to explain the sequential appearance of polytene chromosome puffs in insect larval salivary glands in response to the steroid hormone 20-hydroxyecdysone (hereafter referred to as ecdysone) [1]. Thus, steroid-triggered transcriptional targets have been historically divided into two groups based on their sequence of expression and their dependence on new protein synthesis: “early” or primary response genes and, “late” or secondary response genes. Many primary response genes encode transcription factors that regulate transcription of secondary response genes, which, in turn, encode critical regulators that direct biological responses [2]. However, it remains poorly understood how systemic steroid hormone pulses are refined into distinct transcriptional cascades that direct tissue-specific responses.

Pulses of ecdysone trigger the major developmental transitions during *Drosophila* development. At the onset of metamorphosis, two sequential pulses transform a crawling larva into an immature adult within half a day. The first pulse, at the end of larval development, triggers puparium formation and initiates prepupal development. Approximately 12 hours later, the prepupal pulse triggers head eversion and initiates pupal development. This transformation from larva to pupa requires the rapid and parallel execution of two major types of processes: the morphogenesis of small clusters of precursor cells into adult structures and the destruction of obsolete larval tissues. Ecdysone triggers both of these processes in a stage- and tissue-specific manner. Thus, these events at the onset of metamorphosis provide an ideal context within which to study the mechanisms that mediate specificity to systemic pulses of ecdysone.

Our approach is to use unbiased genetic screens to identify mutations that disrupt a subset of tissue-specific responses to ecdysone at the onset of metamorphosis, expecting that these mutations, and the genes they affect, will provide insights into mechanisms that mediate specificity to hormonal pulses. Our primary experimental context is the destruction of larval salivary glands in response to the prepupal pulse of ecdysone. Reverse genetic approaches have led to the identification of three ecdysone early response genes required for this ecdysone-triggered biological response. Loss of *E74A* (FBgn0000567), *E93* (FBgn0264490) or *BR-C* (FBgn0000210) disrupt the ecdysone-induced expression of apoptotic activators *reaper* (*rpr*) (FBgn0011706) and *head involution defective* (*hid*) (FBgn0003997) and result in a persistent salivary gland (PSG) phenotype [3]–[6]. We previously completed a large-scale EMS mutagenesis to identify additional mutations that specifically disrupt this ecdysone-triggered response [7]. Here, we report our analysis of one of these newly identified PSG mutations that maps to the DEAD box RNA helicase *belle* (FBgn0263231).

DEAD box RNA helicases are characterized by the presence of an Asp-Glu-Ala-Asp (DEAD) motif and use ATP to bind or remodel RNA and RNA-protein complexes (ribonucleoprotein (RNP) complexes) [8]. Members of this family of proteins contain a highly conserved helicase core with many characteristic sequence motifs that include binding sites for ATP and RNA. The helicase core is flanked by domains that are thought to allow interactions with other proteins or with the target RNA. *belle* and its orthologs from yeast (*Ded1*) (YOR204W) to humans (*DDX3*) (ENSG00000215301) are thought to be involved in many aspects of RNA metabolism, including pre-mRNA splicing, nuclear RNA export, RNA interference, translational repression and translational initiation. Recent work with the yeast ortholog *Ded1* provides a model for how this family of proteins regulate both translation initiation and repression [9]. Ded1 directly assembles a translational pre-initiation complex which represses translation; after subsequent ATP-hydrolysis, Ded1 releases the RNP complex allowing translation initiation. However, the role of *belle/DDX3* in development remains poorly understood.

We demonstrate that *belle*-dependent translational control regulates a subset of transcriptional cascades triggered by ecdysone during *Drosophila* metamorphosis. We identify the ecdysone early response gene *E74A* as a critical mRNA target of Belle. Thus, *belle*-dependent translation of *E74A* determines whether E74A-dependent targets of the ecdysone transcriptional cascade are properly regulated. One property of this translational control is that E74A protein is expressed in a subset of cells and tissues during metamorphosis even though ecdysone-triggered transcription of *E74A* occurs in most, if not all, cells. Our results also provide insight into mechanisms that mediate the self-limiting behavior of steroid-triggered transcriptional responses.

## Results

### *belle* disrupts a subset of ecdysone-triggered responses during metamorphosis

**Figure 1:**
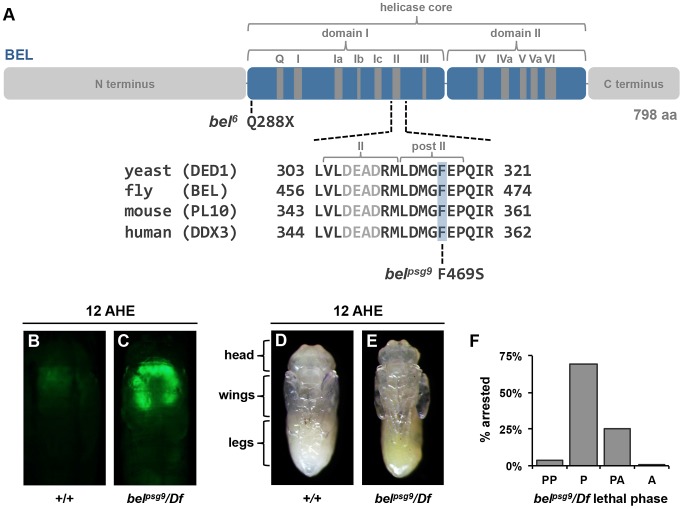
Mutation in *belle* disrupts a subset of ecdysone-triggered responses during metamorphosis. (A) Schematic of conserved motifs in the DEAD-box RNA helicase (adapted from [8]) and sequence alignment of the DEAD-box containing motif II and the *belle/DDX3* family-specific post II region. *bel^psg9^* has a missense mutation that changes a phenylalanine to a serine at residue 469 within the post II region. *bel^6^* has a nonsense mutation at residue 288 introducing an early stop codon. (B–C) Live pupae at 12 AHE with salivary gland specific expression of GFP. Salivary gland GFP (*fkh-GAL4*, *UAS-GFP*) is no longer present in control animals at 12 AHE (B) but persists in similarly staged *bel^psg9^* mutant glands (C), indicating a block in the ecdysone-triggered destruction of this tissue. (D–E) Control (D) and *bel^psg9^* mutant (E) pupae dissected out of their pupal cases 12 hours after head eversion (AHE). Both control and mutant pupae have everted heads and fully extended legs and wings, suggesting that the global ecdysone-induced genetic hierarchy is intact. (F) Lethal phase analysis of *bel^psg9^* hemizygous animals. Most (70%) *bel^psg9^* mutant animals arrest as newly formed pupae (n = 215). PP: prepupae, P: pupae, PA: pharate adults, A: adults eclosed.

To obtain insights into the mechanisms that regulate specificity to systemic pulses of ecdysone, we previously conducted a genetic screen for mutations that disrupt the ecdysone-triggered destruction of larval salivary glands during metamorphosis [7]. Here, we report characterization of one mutation identified in this screen: *psg9*. We used recombination mapping with pairs of dominant markers to estimate the location of the *psg9* locus (method described in [7]) and performed complementation tests with chromosomal deficiencies to further refine its location. This approach placed *psg9* in a molecularly defined region that contained seven candidate genes, including the DEAD box RNA helicase *belle* (Figure S1A). Subsequent complementation tests with available lethal mutations in this region, Sanger sequencing and RNA-interference knockdown of candidate genes, confirmed that the EMS-induced lesion in *psg9* mapped to *belle* (data not shown). *psg9* failed to complement previously reported lethal mutations in *belle*, including the EMS-induced allele *bel^6^* [10]–[12] and the P-element-induced allele *bel^L4740^* [13] (Figure S1B). Sequencing the *belle* open reading frame in *psg9* mutant animals identified a single missense mutation that results in a non-polar to polar substitution (F469S) within a region that is identical among *belle*'s orthologs from yeast to humans (Figure 1A; see “II” and “post II”). Previously reported mutations in either the DEAD box-containing motif II or the *belle/DDX3* family-specific post II region disrupt translation of target mRNAs [9], [14]. Thus, *bel^psg9^* likely disrupts translational initiation of *belle* targets during *Drosophila* development.

*bel^psg9^* mutant (refers to *bel^psg9^/Df* unless otherwise stated) animals showed a strong persistent salivary gland (PSG) phenotype (93%, n = 94) (Figure 1C and Figure S1B), indicating a block in the ecdysone-triggered destruction of this larval tissue. This defect was not due to a general inability to respond to ecdysone. Prior salivary gland-specific responses to ecdysone, like synthesis of glue proteins during the mid-third instar and secretion of gland contents during puparium formation, occur normally in *bel^psg9^* mutant salivary glands (data not shown). In addition, *bel^psg9^* mutant animals pupariate, head evert and extend their legs normally (Figure 1E), indicating that the global ecdysone-induced genetic hierarchy was not affected. Most *bel^psg9^* mutant animals arrest progression of development soon after head eversion as newly formed pupae (Figure 1F). This stage-specific lethal phase during metamorphosis is unique to the *bel^psg9^* allele. Two previously described mutations, *bel^6^* and *bel^L4740^*, have lethal phases early in development without any mutant animals surviving to metamorphosis (Figure S1B). Both alleles are likely strong hypomorphic or null alleles: *bel^L4740^* does not appear to make any protein [13] and we identified a nonsense mutation in *bel^6^* (Q288X) that removes the entire RNA helicase and C-terminal domains (Figure 1A). Accordingly, *bel^psg9^* transheterozygous (*bel^psg9^*/*bel^6^* and *bel^psg9^*/*bel^L4740^*) animals have a similar phenotype to *bel^psg9^* hemizygous animals, arresting after head eversion with a highly penetrant block in the destruction of larval salivary glands (Figure S1B). Thus, *bel^psg9^* disrupts a subset of ecdysone-regulated responses and is required for progression through metamorphosis.

### Ecdysone early-response genes are properly induced

**Figure 2:**
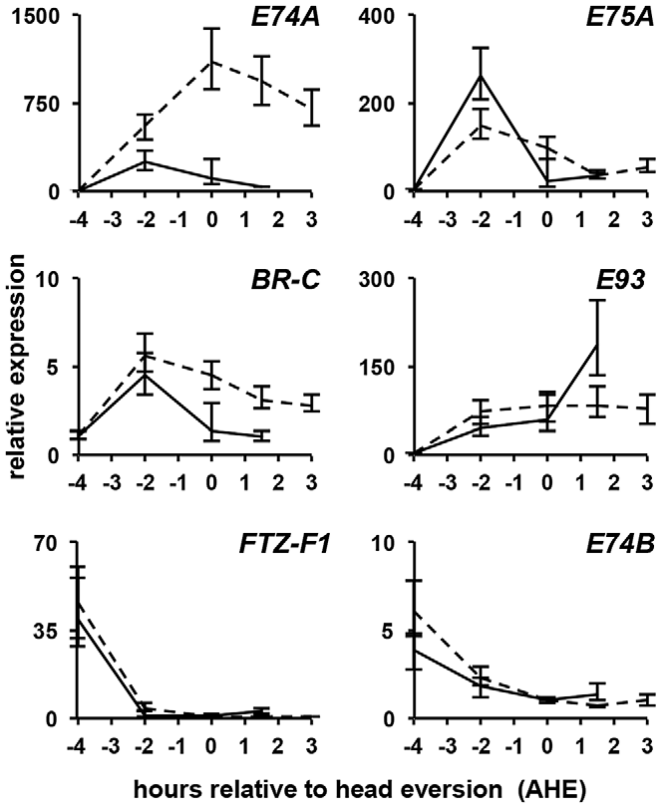
*belle* selectively disrupts regulation of the ecdysone early response gene *E74A*. qPCR analysis of ecdysone hierarchy genes in control (solid lines) and *bel^psg9^* mutant (dashed lines) larval salivary glands. Expression profiles of the ecdysone hierarchy genes *E75A*, *BR-C*, *E93*, *FTZ-F1 and E74B* are relatively normal in *bel^psg9^* mutant salivary glands. In contrast, the early response gene *E74A* is not properly repressed. y-axis plots relative expression, normalized to *rp49*; x-axis plots developmental stage relative to head eversion. Each time point represents three independently isolated salivary gland samples. AHE: after head eversion.

**Figure 3:**
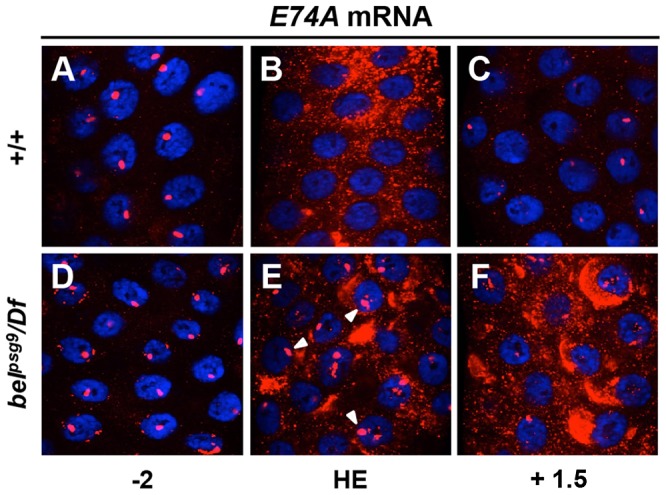
Transcription of *E74A* is not properly repressed in *belle* salivary glands. Fluorescent *in situ* hybridizations for *E74A* mRNA in control (A–C) and *bel^psg9^* mutant (D–F) salivary glands shown in red with DAPI costained nuclei in blue. Two hours before head eversion (−2 AHE), *E74A* mRNA is detected as strong nuclear bands indicative of active transcription at ecdysone-induced chromosomal puffs in both control (A) and mutant (D) glands. In control glands at head eversion (HE) (B), *E74A* mRNA is primarily cytoplasmic and nuclear puff staining is no longer detectable. In contrast, *bel^psg9^* mutant glands at HE (E) show continued nuclear puff staining (arrowheads) and increased cytoplasmic staining. At +1.5 AHE, *E74A* mRNA is barely detectable in control glands (C). Similarly staged mutant glands (F) show continued accumulation of cytoplasmic *E74A* RNA. HE: head eversion.

Given the highly penetrant PSG phenotype in *bel^psg9^* mutant animals, we used the ecdysone-triggered destruction of larval salivary glands as an experimental context for molecular characterization of *belle* function. As a first step towards understanding how *bel^psg9^* disrupts this ecdysone-triggered response, we examined expression of key components of the ecdysone-induced transcriptional hierarchy. Salivary glands were dissected from control and mutant animals staged relative to head eversion (hours after head eversion or AHE) and subjected to quantitative real-time reverse transcriptase PCR (qPCR) analysis. As expected, in control salivary glands, expression of the classical ecdysone early response genes *E74A*, *E75A* (FBgn0000568) and *BR-C*, and the stage-specific ecdysone early response gene *E93*, peak with the prepupal pulse of ecdysone at −2 AHE (Figure 2, solid lines). In *bel^psg9^* mutant salivary glands, these ecdysone early response genes are similarly induced by −2 AHE (Figure 2, dashed lines). Accordingly, RNA *in situ* hybridizations with probes directed to *E74A* mRNA indicate that ecdysone-induced transcription of early response genes was not affected. In both control and *bel^psg9^* mutant glands at −2 AHE, *E74A* RNA staining appears as single bands inside the nucleus, reflecting staining of newly transcribed RNA within ecdysone-induced puffs on polytene chromosomes (Figure 3A and 3D). Thus, transcription triggered by the prepupal pulse of ecdysone was unaffected in *bel^psg9^* mutant salivary glands.

### Ecdysone-induced transcription of *E74A* is not properly repressed

A hallmark of the classical ecdysone early response genes *E74A*, *E75A* and *BR-C* is that their expression increases with rising titers of ecdysone but quickly regresses to basal levels. This self-limiting behavior is evident in salivary glands dissected from control animals, where *E74A*, *E75A* and *BR-C* mRNA return to low levels two hours after their peak expression (Figure 2). In *bel^psg9^* mutant salivary glands, however, *E74A* mRNA expression continues to rise, reaching ∼1,000-fold induction by head eversion and failing to return to pre-pulse levels even three hours later (Figure 2). Salivary gland-specific knockdown of *belle* using RNA interference shows a similar effect on *E74A* mRNA levels (data not shown), suggesting that the effects of *belle* are cell autonomous. The ecdysone-dependent repression of mid-prepupal genes *ftz-f1* (FBgn0001078) and *E74B* occurs normally in *bel^psg9^* mutant glands (Figure 2), suggesting that the ecdysone regulated transcriptional repression machinery is unaffected. We then used RNA *in situ* hybridizations to further characterize the *E74A* mRNA defects in *bel^psg9^* mutant salivary glands. In control glands, the ecdysone-induced nuclear puff staining at −2 AHE (Figure 3A), disappears by head eversion when *E74A* RNA is predominantly cytoplasmic (Figure 3B). In *bel^psg9^* mutant glands at head eversion, however, *E74A* RNA is still detected in strong nuclear puffs (Figure 3E; see arrowheads), suggesting that transcription of *E74A* and the associated polytene chromosome puffs fail to regress. These mutant glands show increased accumulation of cytoplasmic *E74A* RNA (Figure 3E), a likely consequence of continued transcription. By +1.5 AHE, *bel^psg9^* mutant salivary glands show further accumulation of cytoplasmic *E74A* RNA but decreased nuclear puff staining (Figure 3F). The decrease in nuclear puff staining at +1.5 AHE likely reflects reduced transcription due to diminishing systemic levels of ecdysone at this stage [15]. This data demonstrates that the ecdysone-dependent transcription of *E74A* is not properly repressed in *bel^psg9^* mutant glands, resulting in increased levels of *E74A* transcripts. The accumulating cytoplasmic RNA indicates that *bel^psg9^* does not affect nuclear export of *E74A* mRNA. In addition, given that the *E74A* qPCR primers span an intron, *bel^psg9^* does not appear to disrupt *E74A* pre-mRNA splicing. *E75A* and *BR-C* mRNA expression profiles show a relatively minor effect on their post-ecdysone pulse levels in *bel^psg9^* mutant glands (Figure 2). Thus, *bel^psg9^* has a strong and specific effect on the regulation of *E74A* transcription.

### *belle* directly regulates translation of E74A

**Figure 4:**
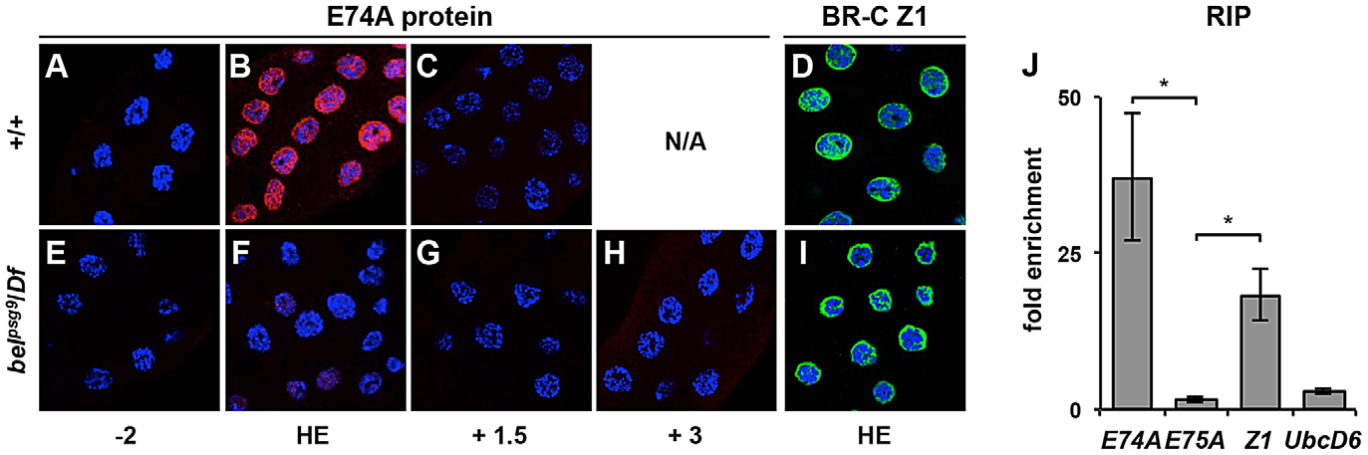
Belle directly regulates *E74A* mRNA translation. (A–I) Salivary glands dissected from animals staged relative to head eversion and stained with antibodies directed to E74A protein shown in red (A–C and E–H) or to BROAD Z1 protein in green (D, I) with DAPI costained nuclei in blue. (A–C) In control salivary glands, E74A protein is detected at head eversion (HE)(B), but not 2 hours before HE (A) or 1.5 hours AHE (C) (N/A: control glands are too fragile to dissect after +1.5 AHE). (E–H) In *bel^psg9^* mutant salivary glands, E74A protein is barely detectable at stages before, during or after head eversion. (D–I) In contrast, both control and *bel^psg9^* mutant salivary glands express BROAD Z1 protein at head eversion. (J) Ribonucleoprotein (RNP) immunoprecipitation (RIP) experiments with BEL-GFP fusion protein, followed by qPCR analysis for target mRNAs. *E74A* and *BR-C Z1* transcripts showed significant (37-fold and 18-fold, respectively) enrichment in BEL-GFP containing RNP complexes, while *E75A* and *UbcD6* transcripts did not show significant enrichment. Data represent average qPCR results from three independent RIP experiments; asterisks indicate p-values <0.05. HE: head eversion, *Z1*: *BR-C Z1*.

The failure to repress *E74A* transcription observed in *bel^psg9^* salivary glands demonstrates that transcriptional regression of an ecdysone early response gene can be selectively disrupted *in vivo*. This result is reminiscent of organ culture studies describing effects of the protein synthesis inhibitor cycloheximide on ecdysone-induced puffs in salivary gland polytene chromosomes [16]. These classical experiments demonstrated that cycloheximide did not affect induction of ecdysone early response puffs but blocked their regression, leading Ashburner to propose a model in which the protein products of early response genes repress their own transcription [1]. Based on this model, we predicted that *belle* was required for *E74A* mRNA translation and that failure to repress *E74A* transcription was due to lack of E74A protein. To test this hypothesis, we used antibodies directed to E74A protein to stain salivary glands dissected from appropriately staged control and *bel^psg9^* mutant pupae. In control salivary glands, E74A protein is expressed as a pulse at head eversion, with little expression 2 hours earlier or 1.5 hours later (Figure 4A–4C). As predicted, E74A protein is barely detectable in *bel^psg9^* mutant salivary glands at head eversion (Figure 4F). Even at +3 AHE, when control salivary glands are too fragile to dissect and persistent *bel^psg9^* mutant salivary glands have very high levels of *E74A* mRNA, E74A protein is still not detected (Figure 4H). Consistent with these observations, western blots of whole animal extracts from control and *bel^psg9^* mutant pupae at head eversion showed a strong reduction in E74A protein (Figure S2A). Importantly, *belle* does not disrupt translation of other ecdysone-induced early response genes. Antibodies directed to BR-C common and Z1 specific isoforms show expression in control and *bel^psg9^* mutant salivary glands (Figure 4D, 4I and data not shown). The cytoplasmic localization of Belle protein in both control and *bel^psg9^* mutant salivary glands (Figure S2B and S2C) is consistent with a role in translational control. These results indicate that *belle* is required for translation of E74A protein.

We then performed RNA-binding protein immunoprecipitation (RIP) experiments to test if *E74A* mRNA is a direct target of Belle protein. We used extracts from appropriately staged animals carrying a GFP exon trap in *belle*: the resulting BEL-GFP fusion protein in these animals (*bel^GFP-ZCL1911^*) is driven under the control of *belle*'s own promoter [17], generates a protein of expected size and does not disrupt *belle* function (Figure S3A–S3B and data not shown). Antibodies directed to GFP were used to immunoprecipitate (IP) BEL-GFP containing ribonucleoprotein (RNP) complexes which were analyzed by RT-PCR (semi-quantitative) or qPCR (quantitative) to test for enrichment of associated mRNAs. Western blots showed effective pulldown of BEL-GFP from lysates (Figure S3C), subsequent semi-quantitative RT-PCR showed that anti-GFP antibodies preferentially immunoprecipitate *E74A* mRNA from lysates containing BEL-GFP (Figure S3D). For quantitative RIP analysis, lysates from *bel^GFP-ZCL1911^* animals were immunoprecipitated and analyzed by qPCR for relative enrichment of target RNAs. We chose the ecdysone early response gene *E75A* and the house-keeping gene *UbcD6* (FBgn0004436) as controls because their endogenous levels were about 2-fold higher than those of *E74A* RNA (Figure S3E) and thus were similarly available potential targets of BEL-GFP. Quantitative analysis from three independent RIP experiments showed that BEL-GFP binding to *E74A* transcripts had an average 37-fold enrichment, while *E75A* and *UbcD6* did not show significant enrichment (Figure 3J). Interestingly, *BR-C Z1* mRNA also associates with Belle-containing RNPs even though its translation is not affected in *bel^psg9^* mutant glands. Taken together, our results indicate that *belle* directly regulates translation of *E74A* mRNA.

### E74A protein inhibits ecdysone-triggered E74A transcription

**Figure 5:**
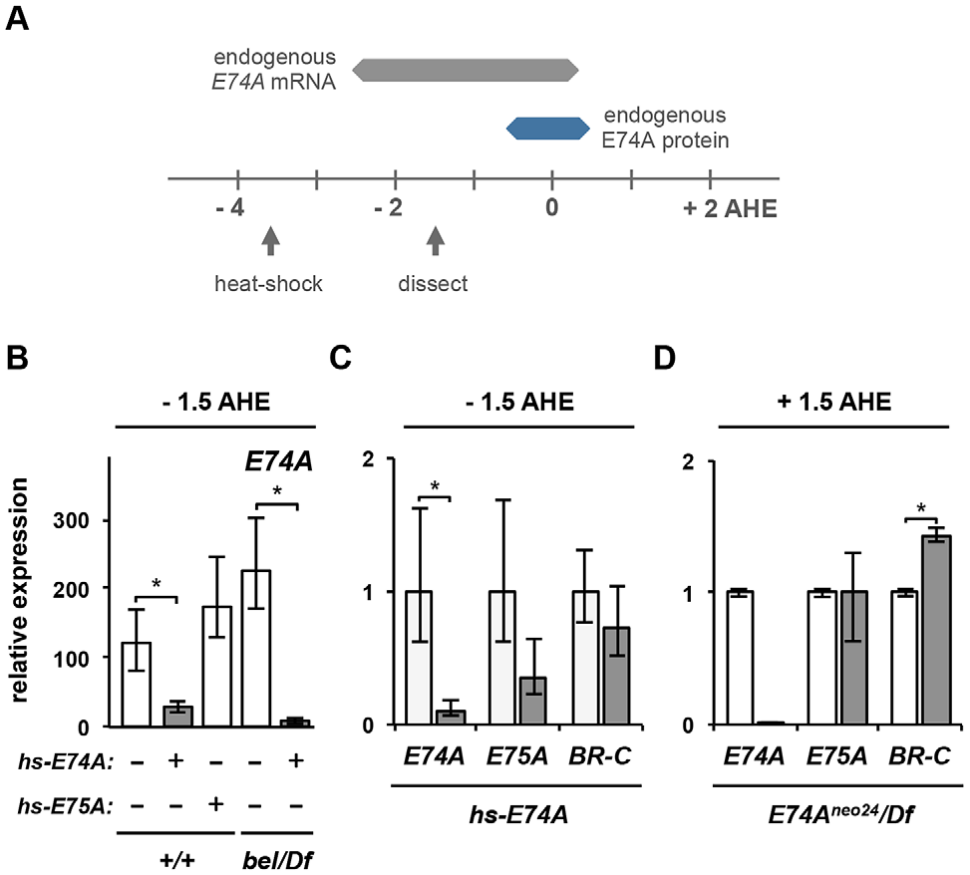
E74A protein is sufficient to inhibit its own ecdysone-induced transcription. (A) Experimental paradigm for expressing E74A protein prior to the prepupal pulse of ecdysone. Expression profile of endogenous *E74A* mRNA (in gray) and E74A protein (in blue) in salivary glands shown relative to hours after head eversion (AHE). Arrows mark timing of the 30 minute heat-shock treatment and the subsequent dissection of larval salivary glands. (B) Precocious expression of E74A protein (gray bars) is sufficient to repress induction of endogenous *E74A* mRNA in control and *bel^psg9^* mutant salivary glands. Control salivary glands show over 100-fold induction of *E74A* in response to the prepupal pulse of ecdysone (first bar from left). In contrast, ectopic expression of E74A protein (second bar), but not E75A protein (third bar), inhibits endogenous *E74A* transcription. Similarly, ectopic E74A protein inhibits the ecdysone-induced expression of *E74A* in *bel^psg9^* mutant salivary glands (fourth and fifth bars). (C) Although precocious expression of E74A protein is sufficient to repress the ecdysone-induced transcription of *E74A*, it has a minor effect on the expression of ecdysone early response genes *E75A* and *BR-C Z1*. Salivary glands dissected from heat-shock-treated control (white bars) and *hs-E74A* carrying (gray bars) animals. (D) *E74A* is not necessary for the regression of other ecdysone early response genes. Salivary glands dissected from control (white bars) and *E74A^neo24^/Df* mutant (gray bars) animals at +1.5 AHE, when endogenous expression of early response genes *E75A* and *BR-C* have regressed to levels prior to the ecdysone pulse. *E74A^neo24^* allele is an RNA null, hence the barely detectable levels of endogenous *E74A* mRNA. y-axis plots relative expression, normalized to *rp49*. Expression ratios in 5B calculated relative to −4 AHE control samples to show induction in response to the prepupal pulse of ecdysone. All samples are in triplicate; asterisks indicate p-values <0.05. *bel/Df*: *bel^psg9^/Df*. AHE: after head eversion.

If accumulation of *E74A* mRNA in *bel^psg9^* mutant salivary glands is due to the absence of E74A protein, then ectopic E74A protein should be sufficient to repress the ecdysone-induced transcription of *E74A*. To test this hypothesis, we used a transgenic construct expressing the *E74A* open reading frame, without its endogenous untranslated regions (UTR), under the control of the heat-shock promoter [18]. The expectation was that *hs-E74A*-derived transcripts, removed from their normal translational regulatory context, would circumvent the requirement for *belle*. Control and *bel^psg9^* mutant animals carrying the *hs-E74A* transgenic construct were heat-shocked at −3.5 AHE and allowed to develop for two hours until −1.5 AHE (experimental paradigm illustrated in Figure 5A). Salivary glands do not express E74A protein at −1.5 AHE (Figure S4A); however, heat-shocked control and *bel^psg9^* mutant animals carrying *hs-E74A* express E74A protein in salivary glands (Figure S4B and S4C). Ecdysone-induced expression of endogenous *E74A* mRNA (distinguished from *hs-E74A*-derived RNA with 5′UTR specific qPCR primers) is at or near its highest levels at −1.5 AHE; however, ectopic expression of E74A protein at this stage inhibits endogenous *E74A* mRNA expression in salivary glands (Figure 5B). This inhibition was specific to E74A protein because a similar experiment with heat-shock-induced expression of E75A protein does not repress *E74A* transcription (Figure 5B). In *bel^psg9^* mutant animals, expression of E74A protein is also sufficient to inhibit ecdysone-induced transcription of *E74A* (Figure 5B). These results suggest that, as predicted by Ashburner, the protein product of the early response gene *E74A* represses its own ecdysone-induced transcription.

Implicit in Ashburner's original model was that protein products of each early gene repress their own transcription [1]. Experimental manipulations of gene dosage performed by Walker and Ashburner are consistent with the idea that E74A and E75A early genes are auto-regulated: adding a copy of the 74EF-75B region results in puffs that regress more rapidly, while removing a copy of the region results in puffs that take longer to regress [19]. Accordingly, E74A and E75A proteins have been shown to bind their own promoters [20], [21]. However, in addition to binding their own ecdysone-induced puffs on polytene chromosomes, E74A and E75A proteins show strong binding to each other's puffs [20], [22], raising the possibility of an alternate model in which early response genes repress each other. To distinguish between these auto-inhibitory and cross-inhibitory models, we measured expression of the three classical early response genes (*E74A*, *E75A* and *BR-C*) in salivary glands dissected from *hs-E74A*-treated and from *E74A* mutant pupae. Although expression of E74A protein prior to the prepupal pulse of ecdysone has a dramatic effect on induction of *E74A* mRNA, it does not have a significant effect on either *E75A* or *BR-C* induction (Figure 5C). Conversely, salivary glands dissected from *E74A*-specific RNA null mutant (*E74A^neo24^/Df*) pupae at +1.5 AHE showed no effect on the regression of *E75A* expression and a minor effect on regression of *BR-C* (Figure 5D). Thus, our data suggests that although cross-inhibitory regulation may occur, auto-inhibition appears to be the primary mechanism for the self-limiting behavior of ecdysone early response genes like *E74A*.

### *belle* and *E74A* selectively disrupt ecdysone-triggered expression of *rpr* and *hid*

**Figure 6:**
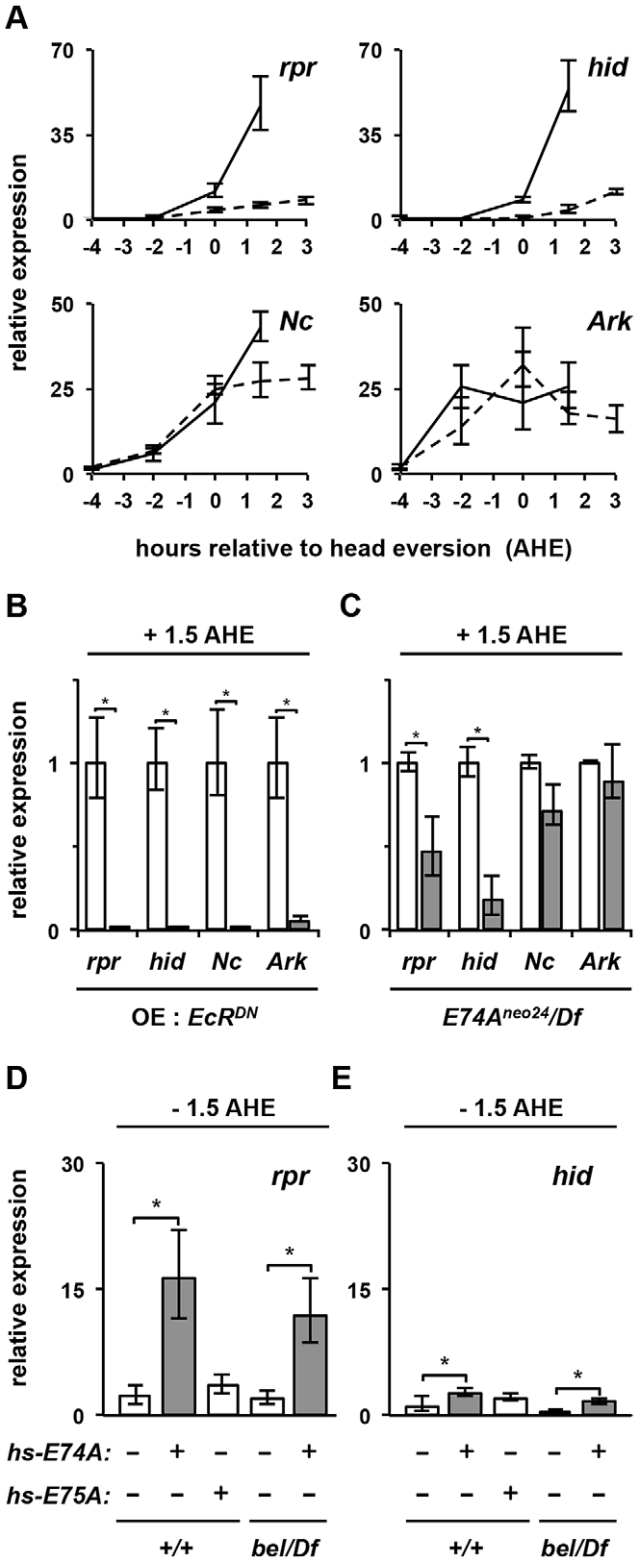
Expression of E74A protein–dependent ecdysone late-response genes is disrupted in *belle* glands. (A) *bel^psg9^* is required for ecdysone-induced expression of death activators *rpr* and *hid*, but not *Nc* and *Ark*. x-axis plots timecourse of salivary glands dissected from control (solid lines) and *bel^psg9^/Df* (dashed lines) animals staged relative to head eversion (AHE) (salivary glands cannot be recovered from control animals after +1.5 AHE). (B) Ectopic expression of dominant negative ecdysone receptor prior to the prepupal pulse of ecdysone blocks expression of death regulators *rpr*, *hid*, *Nc*, and *Ark* in salivary glands. Control (white bars) and *OE: EcR^DN^* (*UAS-GAL4/UAS-EcR^F645A^; hs-GAL4/+*) (gray bars) preupupae were heat-shocked four hours before head eversion (−4 AHE), re-staged at head eversion and salivary glands were dissected at +1.5 AHE, a stage when these death genes are maximally expressed. (C) *E74A* is required for ecdysone-induced expression of death activators *rpr* and *hid*, but not *Nc* and *Ark*. Relative expression of death regulators in salivary glands dissected from control (white bars) or *E74A^neo24^/Df* mutant (gray bars) animals. (D–E) Precocious expression of E74A protein (gray bars) is sufficient to significantly induce expression of *rpr* (D) and *hid* (E) in salivary glands (experimental paradigm as Figure 5A). (D) Ectopic expression of E74A protein in control (second bar from left) and *bel^psg9^* mutant (fifth bar from left) animals show significant induction of *rpr*. In contrast, ectopic expression of E75A protein (third bar from left) does not show any effect. (E) Similar experiments measuring *hid* expression show minor but statistically significant changes. y-axis plots relative expression, normalized to *rp49*. Expression ratios in 6D and 6E calculated relative to −4 AHE control samples to show induction in response to the prepupal pulse of ecdysone. All samples are in triplicate; asterisks indicate p-values <0.05. *bel/Df*: *bel^psg9^/Df*. AHE: after head eversion.

To demonstrate that *belle* disrupts *E74A*-dependent ecdysone responses, we characterized the ecdysone-triggered death response in *bel^psg9^* mutant salivary glands. The destruction of larval salivary glands is triggered, at least in part, by expression of death activators *rpr* and *hid* in response to the prepupal pulse of ecdysone [23]. As expected, *rpr* and *hid* are induced in control salivary glands by the prepupal pulse of ecdysone, showing expression beginning at head eversion (0 AHE) and reaching 50-fold induction 1.5 hours later (+1.5 AHE) (Figure 6A). Salivary glands from *bel^psg9^* mutant animals, however, do not show induction of either *rpr* or *hid* (Figure 6A), suggesting that *bel^psg9^* acts upstream of death activator expression in the ecdysone-triggered transcriptional cascade. Expression of the *Drosophila* Inhibitor of Apoptosis Protein (*diap1*) (FBgn0260635) is not affected in mutant salivary glands (data not shown). In addition, *bel^psg9^* does not affect the ecdysone-induced expression of other death genes at this stage. The apical caspase *Nc* (FBgn0026404) and the caspase adaptor *Ark* (FBgn0263864)(homologs of the vertebrate *caspase-9* and *Apaf-1*, respectively) are also induced during the death response in salivary glands [4], [24]–[26]. However, *Nc* and *Ark* are properly induced in *bel^psg9^* mutant salivary glands (Figure 6A), suggesting that *bel^psg9^* selectively disrupts expression of *rpr* and *hid* in response to the prepupal pulse of ecdysone. Expression of a dominant negative ecdysone receptor immediately prior to head eversion blocks salivary gland-specific expression of *rpr*, *hid*, *Nc* and *Ark* (Figure 6B), confirming that these genes are induced in response to the prepupal pulse of ecdysone. Three components of the ecdysone hierarchy have been shown to disrupt the death response in larval salivary glands: *BR-C[rbp^5^]*, *E74A* and *E93* [3]–[5]. Of these, *BR-C[rbp^5^]* and *E93* affect expression of *Nc* and/or *Ark* [4], [5], [27]. On the other hand, salivary glands dissected from *E74A*-specific null mutant (*E74A^neo24^*/Df) pupae at +1.5 AHE show a significant reduction in levels of both *rpr* and *hid*, without any effects on expression of *Nc* or *Ark* (Figure 6C). Thus, like *bel^psg9^*, *E74A* selectively regulates *rpr* and *hid* expression, demonstrating that the effects of *bel^psg9^* during the ecdysone-induced death response in salivary glands are mediated primarily by *belle*'s effects on E74A protein. Moreover, *E74A* mutant animals have very similar phenotypes to those described above for *bel^psg9^*, with mutant animals dying after head eversion and showing a highly penetrant PSG phenotype (data not shown and [3], [7], [28]). Together, our data suggest that *bel^psg9^* disrupts expression of E74A-dependent ecdysone late genes.

### E74A protein rescues *rpr* expression in *belle* glands

To demonstrate that *belle*'s effects on *E74A*-dependent targets are mediated by E74A protein, we expressed E74A protein in control and *bel^psg9^* mutant salivary glands using the *hs-E74A* transgenic construct (using paradigm described in Figure 5A). Control salivary glands have very low levels of *rpr* and *hid* mRNA at −1.5 AHE; in contrast, precocious expression of E74A protein resulted in a significant induction of *rpr* (Figure 6D). This effect was specific to E74A protein, since the expression of E75A protein under the same conditions did not have any effect on *rpr* expression (Figure 6D). Importantly, in *bel^psg9^* mutant glands, like in control glands, E74A protein was sufficient to precociously induce *rpr* (Figure 6D). Although *E74A* is necessary for *hid* expression (Figure 6C), precocious E74A protein had a reduced but significant effect on *hid* expression in both control and mutant glands (Figure 6E), suggesting that ecdysone-dependent regulation of *hid* is more complex than that of *rpr*. Our attempts to use *hs-E74A* to experimentally rescue the PSG phenotype in *bel^psg9^* mutant animals failed, suggesting that E74A protein alone is not sufficient for tissue destruction. Alternatively, Belle may have other targets required for a proper death response in salivary glands. Importantly, however, our expression analysis results demonstrate that E74A protein is sufficient to rapidly rescue the loss of ecdysone-induced expression of *rpr* in *bel^psg9^* mutant glands.

### Translational, not transcriptional, control determines E74A protein distribution

**Figure 7:**
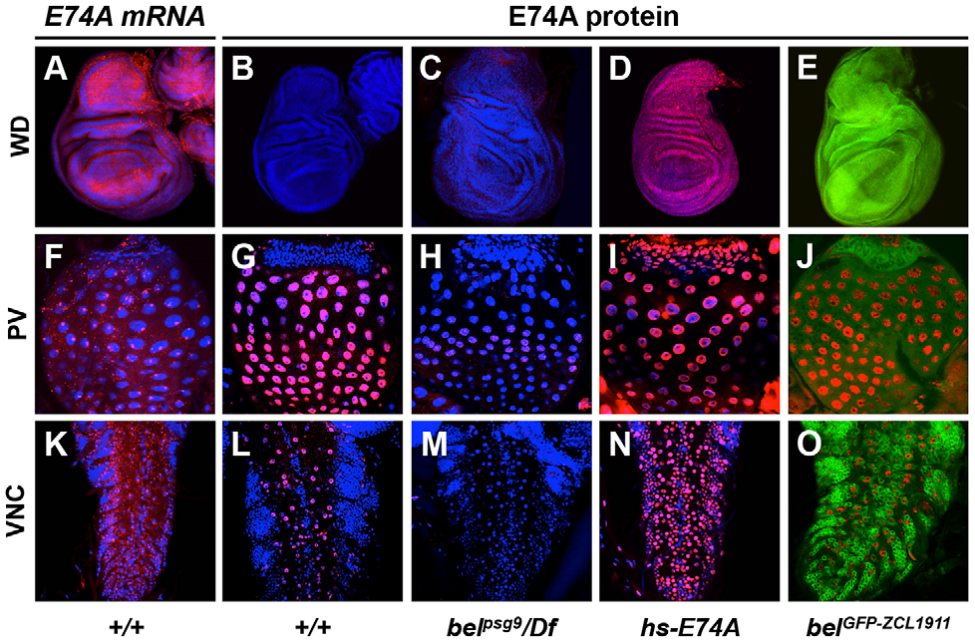
Translational control determines spatial distribution of E74A protein. Tissues at the onset of metamorphosis were dissected and imaged to detect *E74A* mRNA (in red, first column), E74A protein (in red, second through fifth columns) or Belle protein (in green, fifth column). Although ecdysone-induced transcription of *E74A* mRNA appears to be ubiquitously expressed (first column), E74A protein is not (second column). This spatial distribution of E74A protein is disrupted in *bel^psg9^* mutant animals (third column) and becomes ubiquitous after expression of E74A protein from the *hs-E74A* transgene (fourth column). E74A protein is detected in the large cells of the proventriculus (G) and *repo*-positive glial cells in the ventral nerve cord (L and data not shown), but absent in the entire imaginal wing discs (B), the imaginal ring of the proventriculus (G) and neurons in the ventral nerve cord (L). Endogenous Belle protein, visualized with the *bel^GFP-ZCL1911^* exon trap, is expressed in all cells including those that do not express E74A protein (fifth column). Staining with antibodies directed to Belle protein show identical expression patterns (data not shown). For *in situ* hybridizations, tissues were dissected from animals a few hours before puparium formation (clear gut stage) when *E74A* mRNA levels are their highest. For E74A protein and BEL-GFP imaging, tissues were dissected at puparium formation when E74A protein levels are at their highest. DAPI costained nuclei in blue. WD: imaginal wing discs, PV: proventriculus, VNC: ventral nerve cords.

Translational control is a critical mechanism for ensuring proper distribution and subcellular localization of proteins during development [29]. To understand why the ecdysone early response gene *E74A* was under *belle*-dependent translational control, we examined tissue-specific distribution of *E74A* mRNA and protein in response to the late larval pulse of ecdysone at the onset of metamorphosis. All tissues examined showed ubiquitous expression of *E74A* mRNA (wing imaginal discs, proventriculus and ventral nerve cord shown; Figure 7, first column). In contrast, E74A protein showed distinct cell- and tissue-specific expression patterns (Figure 7, second column). Imaginal wing discs, for example, do not show any E74A protein expression despite robust expression of *E74A* mRNA (Figure 7B), while the proventriculus (PV) and ventral nerve cord (VNC) showed E74A protein expression in a subset of cells (Figure 7G and 7L). Consistent with our observations, a previous study reported that in one tissue (the proliferation zone in the brain at the onset of metamorphosis), E74A protein was not detected despite presence of *E74A* mRNA [30], suggesting that *E74A* mRNA was under translational control. Importantly, we showed *bel^psg9^* mutant tissues disrupted E74A protein expression in all tissues examined (Figure 7, third column), demonstrating that *belle*-dependent translational control regulates E74A protein in most, if not all, tissues. Moreover, heat-shock treatment of animals carrying the *hs-E74A* construct is sufficient to express E74A protein in cells that normally do not express it (Figure 7, fourth column). Belle protein is ubiquitously expressed in all tissues examined (Figure 7, fifth column, and data not shown), suggesting that the distribution of Belle alone does not determine where *E74A* mRNA is translated. Cells that do not express E74A protein appear to have higher levels of Belle; however, differences in levels of Belle protein are not sufficient to explain the spatial distribution of E74A protein. For example, reducing the level of Belle protein in VNC neurons or in wing discs does not alter the ability to translate *E74A* mRNA (data not shown). Taken together, these results indicate that regulation of translation, not of transcription, determines the tissue-specific distribution of E74A protein in response to systemic pulses of ecdysone. The mechanisms that regulate Belle-dependent translation of *E74A* mRNA in a cell-type- and tissue-specific manner, however, are yet to be determined.

## Discussion

**Figure 8:**
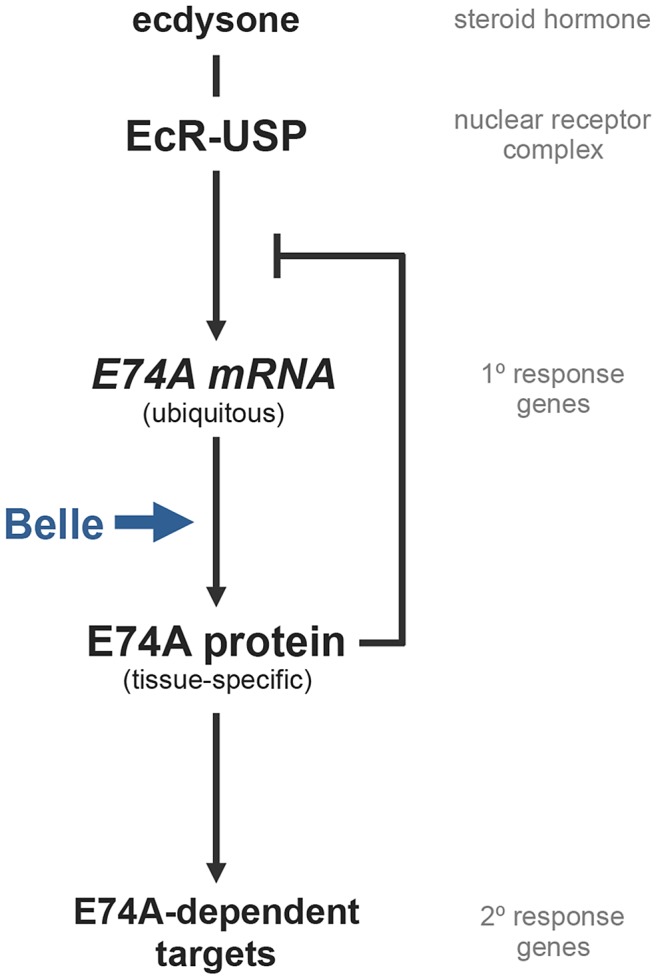
Translational control by Belle regulates E74A-dependent ecdysone-triggered responses. A model of how Belle protein regulates a subset of ecdysone-triggered transcriptional responses. E74A protein has two functions: to induce expression of E74A-dependent target genes and to inhibit its own ecdysone-induced transcription. Given that Belle is required for translation of *E74A* mRNA, Belle activity effectively regulates E74A-dependent targets within the ecdysone-triggered transcriptional cascade. Moreover, Belle-dependent translational control of a ubiquitous transcriptional target of ecdysone like *E74A* occurs in a tissue-specific manner, adding a novel regulatory layer to steroid hormone signaling. Thus, Belle-dependent translational control helps refine global ecdysone signals into distinct tissue-specific transcriptional responses.

Systemically released steroid hormones trigger transcriptional cascades that ultimately direct tissue-specific biological responses. This work demonstrates that translational control mechanisms play a critical role in refining steroid-triggered transcriptional responses (Figure 8). The DEAD box RNA helicase *belle* directly and selectively regulates translation of the ets transcription factor and classical ecdysone early response gene, *E74A*. As a result, in *belle* mutant cells, components of the ecdysone transcriptional cascade downstream of *E74A* are not regulated properly. In addition to regulating these *E74A*-dependent ecdysone late response genes, E74A protein also inhibits its own ecdysone-induced transcription. Moreover, although *E74A* is a ubiquitous transcriptional target of ecdysone signaling, E74A protein is only expressed, in a *belle*-dependent manner, in a subset of cells. Thus, one property of this *belle*-dependent translational control is the ability to provide spatial specificity to systemic pulses of ecdysone.

*belle/DDX3* DEAD box RNA helicases regulate many aspects of RNA metabolism, including splicing, nuclear export and translation; however, the mutation we identified specifically disrupts *belle*'s ability to regulate translation. The lesion in *bel^psg9^* affects the *belle/DDX3* family-specific post II region. The adjacent DEAD box-containing motif II is critical for ATP-hydrolysis-dependent release of translationally repressed RNP complexes [8]; thus, a mutation in the yeast Ded1 DEAD box acts as a strong repressor of translation when overexpressed [9]. Similarly, previously identified mutations in the post II region in *Ded1* also disrupt translation [14]. The requirement for both motif II and post II regions in translation initiation suggests these regions may act together to regulate translation. One model suggests that changes in the post II region may alter the position of the DEAD box, affecting the ability to hydrolyze ATP [31]. Importantly, however, although E74A protein is barely detectable in *bel^psg9^* mutant animals, suggesting a strong block in translation, pre-mRNA splicing and nuclear export of *E74A* mRNA are not affected. These results indicate that the post II region-dependent activity, although critical for translational control, may not be required for other putative functions of Belle.

Our data suggest that *belle* is a selective regulator of translation. Within the ecdysone genetic hierarchy, for example, *belle*-containing RNPs associate with *E74A* mRNA but not with *E75A* mRNA. There is evidence that Belle/DDX3 proteins preferentially regulate translation of transcripts with long and complex 5′UTRs [32], [33], suggesting that target mRNA selection is mediated through interactions with 5′UTRs. Accordingly, *E74A* mRNA has a very long 5′UTR (1.9 kb) [34], whereas *E75A* mRNA has a much shorter 5′UTR (0.69 kb) [35]. Binding alone, however, does not indicate a role in translation initiation: *belle*-containing RNPs include *BR-C Z1* mRNA, but BR-C Z1 protein is not affected in *bel^psg9^* mutant tissues. Although it is possible that *BR-C Z1* mRNA has redundant translation initiation mechanisms, this result is consistent with results from human cells where some *DDX3* bound mRNAs do not require *DDX3* for translation initiation [36]. It remains to be determined how this functional specificity among *belle/DDX3* targets is regulated.

The selective disruption of *E74A* mRNA translation in *belle* mutant animals provided a unique opportunity to directly test a model proposed by Ashburner nearly 40 years ago to explain the temporal behavior of ecdysone-induced puffs in salivary gland polytene chromosomes. According to this model, the gene products of ecdysone early response puffs have two critical functions: initiate expression of subsets of late response genes and inhibit their own ecdysone-induced expression. Indeed, all three classical early puff loci (2B5[*BR-C*], 74EF and 75B) encode transcription factors which, in turn, regulate ecdysone late response genes [2]. This study shows that E74A protein is required for transcription of a subset of ecdysone-triggered late genes in salivary glands. Importantly, we have demonstrated for the first time that the transcription of an ecdysone early response gene is auto-regulated, fulfilling the second prediction of the Ashburner model. E74A protein is both necessary and sufficient to inhibit its own transcription. In the absence of E74A protein, as in *bel^psg9^* mutant salivary glands, ecdysone-induced transcription fails to regress and transcripts continue to accumulate to very high levels; conversely, ectopic expression of E74A protein inhibits *E74A* transcription in both control and *bel^psg9^* mutant salivary glands. Given that E74A protein can bind *in vitro* to DNA sequences within the E74A locus [20], the effect of E74A protein on *E74A* transcription is likely to be direct. In addition, we observe the effect of ectopic E74A protein on *E74A* transcription in whole animal extracts from control and mutant prepupae (data not shown), indicating that *E74A* auto-regulation is not limited to salivary glands.

We propose that *belle*-dependent translational control provides specificity to ecdysone signaling by regulating a subset of ecdysone early response genes. This conclusion is supported by multiple lines of evidence. First, antibody staining experiments indicate that *bel^psg9^* disrupts translation of *E74A* mRNA but not of *BR-C* mRNA. Second, RNA binding protein immunoprecipitation experiments indicate that Belle protein selectively associates with a subset of ecdysone early genes. Third, auto-regulation of *E74A* transcription is strongly disrupted in *bel^psg9^* mutant salivary glands with minor effects on the other early genes, consistent with a selective translation defect. Fourth, *bel^psg9^* disrupts a subset of ecdysone-triggered responses at the onset of metamorphosis that is different from those of *BR-C* or *E75A* mutant animals, but indistinguishable from those of *E74A* mutant animals. Taken together, these results suggest that among the three classical ecdysone early response genes–*E74A*, *E75A* and *BR-C*–only *E74A* mRNA translation is regulated by *belle*, giving *belle* the ability to determine E74A protein-dependent responses at the onset of metamorphosis.

Translational control of ecdysone-triggered responses provides new insight into mechanisms that refine systemic steroid signals into tissue-specific responses. Current explanations for how global hormonal cues are refined into distinct local responses invoke transcriptional mechanisms that regulate DNA binding properties of nuclear receptors, including availability of nuclear receptor co-factors or accessibility to nuclear receptor binding sites at target genes. Our observations, however, suggest that translational control can determine the spatial readout of ubiquitous transcriptional targets of liganded nuclear receptors, adding a previously unknown regulatory layer to steroid hormone signaling. Determining prevalence of translational control within steroid signaling pathways in other organisms and understanding how this *belle/DDX3*-dependent translational control is regulated are important goals for future studies.

## Materials and Methods

### Stocks and recombination mapping

The following stocks were obtained from the Bloomington *Drosophila* Stock Center: *bel^6^* (FBst0004024), *bel^L4740^* (FBst0010222), *Df(3L)81k19* (FBst0002998), *Df(3R)BSC197* (FBst0009623), D*f(3R)Exel6149* (FBst0007628), *Df(3R)p712* (FBst0001968), *E74A^neo24^* (FBst0010262), *hs-GAL4* (FBst0001799), *UAS-EcR^F645A^* (FBst0006869) and *UAS-GAL4* (FBst0005939). *bel^GFP-ZCL1911^* was obtained from Morin *et al.* 2001 [17]. *bel^psg9^* stock was generated in Wang *et al.* 2008 [7]. Use of *fkh-GAL4*, *UAS-GFP* stock is described in Wang *et al.* 2008 [7]. C. Thummel kindly provided the *hs-E74A* and *hs-E75A* stocks. Recombination mapping of *psg9* was conducted using pairs of dominant markers as described previously [7]. For experiments with *E74A* and *bel^psg9^* hemizygous animals described in this report, we used the *Df(3L)81k19* and *Df(3R)Exel6149* chromosomal deficiencies, respectively.

### Developmental staging

Animals were raised on cornmeal molasses media with granulated yeast at 25°C. For developmental staging during metamorphosis, animals were either synchronized at puparium formation (white prepupae) or at pupation (head eversion), placed on damp black filter paper in a Petri dish and aged appropriately at 25°C. For time points before head eversion, animals were staged at puparium formation and aged based on the average duration of prepupal development (time from white prepupae to head eversion). For staging late third instar larvae at the peak of the late larval ecdysone pulse, we used standard “blue food” technique to pick “clear gut” larvae [37]. For heat shock treatments, staged animals were placed on Parafilm-sealed grape agar plates and submerged in a 37.5°C water bath for 30 minutes. Lethal phases were assigned based on the staging criteria described by Bainbridge and Bownes [38]: P1–P3 for prepupae (PP), P4–P9 for pupa (P), P10–P15 for pharate adult (PA), and eclosed adults (A).

### Quantitative RT–PCR

Each sample was prepared by extracting RNA from 16 salivary glands dissected from appropriately staged animals using the RNeasy Plus Mini Kit (Qiagen). cDNA was synthesized from 400 ng of total RNA using the SuperScript III First-Strand Synthesis System with Oligo(dT)_20_ primers (Invitrogen). Quantitative RT-PCR was performed using a Roche 480 LightCycler with the LightCycler 480 DNA SYBR Green I Master kit (Roche). Cycling was performed with an annealing temperature of 59°C and a 6 second extension time; 3–5 ten-fold dilutions of pooled cDNA from samples spanning metamorphosis were used to calculate the amplification efficiency of primer pairs in each experiment. Roche LightCycler 480 Software (Version 1.5) was used to calculate cycle threshold values and melt curve analysis for each reaction. Relative Expression Software Tool (REST) was used to calculate relative expression and standard error [39]. REST uses an integrated randomization and bootstrapping method to calculate confidence intervals and p-values for relative expression ratios. Given that REST calculates standard error based on a confidence interval centered on the median, it reflects the asymmetrical tendencies of the data. Control and experimental condition were analyzed simultaneously with three independent biological samples for both reference (*rp49*) (FBgn0002626) and target genes. Expression ratios were normalized relative to the lowest point in each expression profile or to the paired control (except in Figure 5B, Figure 6D and 6E where control and experimental samples are normalized to −4 AHE control samples that were run simultaneously). Primer sequences are listed in Table S1.

### RNA *in situ* hybridizations

Probe synthesis, tissue preparation, hybridization, and probe detection were carried out according to Wilk *et al.* 2010 [40]. Digoxigenin-labeled probes were synthesized by PCR amplification of *E74A* cDNA using *E74A* 5′UTR-specific primers (*E74A-UTR* F 5′-AGAAATCTCGCTGTTCAAGTGG, *E74A-UTR* R 5′-GCGGCCAAGCA AATACAACAA C). To detect probes after hybridization, tissues were incubated overnight at 4°C in 1∶400 HRP-conjugated mouse monoclonal α-DIG antibody (Jackson Immuno-Research Labs), followed by incubation in 1∶50 Cy3 tyramide conjugates (Perkin Elmer). Nuclei were counterstained with DAPI. Stained tissues were mounted on slides with Vectashield (Vector Laboratories). Tissues from *E74A* mRNA null (*E74A^P[neo]^/Df*) mutant animals do not show staining, demonstrating specificity of probes to *E74A* mRNA (data not shown).

### Immunofluorescence

Dissected tissues from appropriately staged animals were fixed and immunostained using standard methods as described previously [41]. Primary antibodies: 1∶50 mouse α-BR-C Z1, (Developmental Studies Hybridoma Bank), 1∶200 rabbit α-BEL (a generous gift from P. Lasko), 1∶10 mouse α-E74A 11C9, 6C5.3 (a generous gift from C. Thummel). Secondary antibodies: 1∶200 Cy3 α-mouse, 1∶200 Cy3 α-rabbit (Jackson Immuno-Research Labs), 1∶200 AlexaFluor 488 α-mouse/rabbit, (Invitrogen). Stained tissues were mounted on slides with Vectashield (Vector Laboratories).

### Microscopy and image capture

Lethal phase and persistent salivary gland images were obtained and captured using an Olympus SZX16 stereomicroscope coupled to an Olympus DP72 digital camera with DP2-BSW software (Olympus). RNA in situ hybridizations and immunofluorescence images were taken on an Olympus FLUOVIEW FV1000 confocal microscope with FV10-ASW software.

### RNA immunoprecipitation

In a 1.5 ml microcentrifuge tube, 40 appropriately staged *bel^GFP-ZCL1911^* or control whole animals were flash frozen in 50 µl of LB150 (150 mM KCl, 20 mM HEPES, pH 7.4, 1 mM MgCl_2_, 0.1% Triton X-100, 1× Complete Mini–Protease Inhibitors (Roche), 0.04 u/µl RNasin – RNase inhibitors (Promega)). Samples were homogenized and 950 µl LB150 was added to each sample. Cleared extracts were transferred to fresh tubes. Immunoprecipitation (IP) using the direct method was performed with Dynabeads Protein G (Invitrogen) as recommended by the manufacturer. 5 µl rabbit α-GFP (Torrey Pines) was added to antibody (+) samples. Antibody (−) samples received no antibody. All washes were conducted using PBST/BSA (1× PBS, 0.5% BSA, 0.1% Triton X-100, 0.04 u/µl RNasin in DEPC treated H_2_0). After the elution of BEL-GFP-containing RNPs, samples were either subjected to RNA isolation or protein isolation depending on the downstream application. For qualitative assessment by RT-PCR, primer sets specific to *E74A* mRNA were amplified in immunoprecipitated samples and amplicons were analyzed by agarose gel electrophoresis. Gel images were captured using a UVP BioDoc-It system with UV transilluminator (UVP). For quantitative assessment by qPCR, three independent BEL-GFP lysates from −2 AHE were split into two fractions. The first fraction received anti-GFP (+). The second fraction did not (−). Upon elution IPed samples underwent RNA isolation, cDNA synthesis and were analyzed by qPCR. To determine the copy number of each target gene, absolute quantification using LightCycler 480 Software (Version 1.5) (Roche) was performed by analyzing 7 ten-fold dilutions of an amplicon with a known concentration in parallel to immunoprecipitated samples of an unknown concentration. For *E74A*, *E75A*, *Z1* and *Ubcd6* enrichment was calculated by dividing the copy number in the (+) fraction over the (−) fraction.

### Western blotting

10 whole animals were homogenized on ice in 150 µl of hi-salt lysis buffer (25 mM HEPES pH 7.4, 300 mM NaCl, 1.5 mM MgCl_2_, 1 mM EGTA, 0.5% Triton X-100, 50 mM β-glycerophosphate, 50 mM NaF, 1 mM Na_3_ V0_4_, 5 µg/ml Pepstatin A, 1 mM DTT, 5 µg/ml Aprotinin, 200 µM PMSF, 10 µg/ml Leupeptin, 4 nM Microsystin). Samples containing equal amounts of protein in 4× sample buffer (1.0 M Tris-HCl pH 6.8, 8% SDS, 40% Glycerol, 20% 2-Mercaptoethanol) were separated on a 6–10% SDS-PAGE gel and were blotted by standard methods. Membranes were blocked with 5% BSA in PBST (1× PBS, 0.1% Triton X-100) and incubated with diluted primary antibodies overnight at 4°C. Primary antibodies were diluted at 1∶1000 rabbit α-GFP (Torrey Pines), 1∶1000 rabbit α-β-actin (Cell Signaling Technologies), 1∶3000 mouse α-E74A (a gift from C. Thummel). Secondary antibodies were diluted to 1∶30,000 α-rabbit IgG alkaline phosphatase linked whole antibody (GE Healthcare) and α-mouse IgG alkaline phosphatase antibody (Sigma). Membranes were developed with ECF substrate (GE Healthcare) and were imaged using a Storm 840 Scanner (Amersham Bioscience) with ImageQuant TL software version 7.0 (GE Healthcare).

## Supporting Information

Figure S1 Mapping and allelic series of *bel^psg9^*. (A) *psg9* mapped by recombination analysis, complementation tests and Sanger sequencing. Recombination mapping with pairs of dominant markers placed *psg9* in the *Glued* (*Gl*) – *Hairless* (*H*) region of the third chromosome: right of the *Roughened* (*R*) and *Dichaete* (*D*) pair and left of the *Hairless* (*H*) and *Prickly* (*Pr*) pair (see arrows; method described in [7]). Complementation tests with chromosomal deficiencies in this region mapped *psg9*, first to a large cytological deficiency (*Df(3R)p712*), then to a small region defined by two overlapping deficiencies (*Df(3R)BSC197* and *Df(3R)Exel6149*). All publicly available lethal mutations of genes within the minimal region were crossed and multiple alleles of the DEAD-box helicase *bel* failed to complement *psg9*. Sanger sequencing identified lesions in both *bel^psg9^* and *bel^6^*. (B) Lethal phase and persistent salivary gland (PSG) phenotype of *belle* hemizygous and transheterozygous animals. All *bel^psg9^* hemizygous and transheterozygous mutant combinations die after head eversion (as pupae) and have a highly penetrant PSG phenotype. *bel^6^* and *bel^L4740^* hemizygous and transheterozygous animals die prior to puparium formation. PSG was assayed at 24 hours after puparium formation when control animals have 0% PSG (n = 100). Early lethal: embryonic or first larval instar lethal, PP: prepupal lethal, P: pupal lethal, PA: pharate adult lethal, A: adult escapers, n/a - not applicable because mutants do not reach the appropriate stage for PSG assay.
(TIF)

Figure S2 E74A and Belle protein expression in *bel^psg9^* mutants. (A) Western blots of whole animal extracts with antibodies directed to E74A protein. E74A protein is robustly expressed in control (left) but barely detectable in *bel^psg9^* mutant animals (right) at head eversion. β-Actin used as a loading control. (B–C) Larval salivary glands stained with antibodies directed to Belle shown in red with DAPI costained nuclei in blue. Belle protein is cytoplasmic in both control (B) and *bel^psg9^*/*Df* mutant (C) salivary glands at head eversion (HE).
(TIF)

Figure S3 RNA binding protein immunoprecipitation experiments. (A) Schematic depicting the nature of the BEL-GFP protein trap line used (*bel^GFP-ZCL1911^*) [17]. (B) Western blot analysis using antibodies directed to GFP (anti-GFP) detects a BEL-GFP fusion protein of the appropriate size in whole animal lysates (a GFP expressing line used as control). (C) Western blot analysis using anti-GFP detects BEL-GFP in whole animal lysates and in immunoprecipitated samples. As expected, immunoprecipitated BEL-GFP samples from whole animal lysates at −2 AHE enrich BEL-GFP protein. (D) Immunoprecipitated (IP) BEL-GFP containing RNPs detects enrichment of *E74A* transcripts using RT-PCR. Two methods were compared: extracts from control or Bel-GFP animals IP with anti-GFP antibodies (left lanes) or Bel-GFP extracts IP with and without anti-GFP antibodies (right lanes). Both approaches show strong enrichment of *E74A* mRNA in BEL-GFP RNPs. (E) Absolute quantification of mRNA copy number in whole animal lysates at −2 AHE. The copy number for *E74A* mRNA is the lowest compared to the other control genes, further supporting the enrichment in the RNA IP experiments.
(TIF)

Figure S4 Ectopic expression of E74A protein from the *hs-E74A* transgene in salivary glands. Staining with antibodies directed to E74A protein shown in red demonstrates that heat-shock (hs) driven induction of the *hs-E74A* transgene expresses E74A protein in both control (B) and *bel^psg9^*/*Df* mutant (C) salivary glands at a stage (1.5 hours before head eversion) when endogenous E74A protein is not present (A). Ectopic expression paradigm as described in Figure 5A. DAPI costained nuclei in blue.
(TIF)

Table S1 qPCR primer sequences. The first column indicates the forward (F) and reverse (R) primer pair for each target gene. Second column shows sequence for each primer. Each primer pair was designed and validated in this study unless otherwise noted. Source references: (a) [42], (b) [43], (c) [44] and (d) [45].
(DOCX)
