# Supplementary figures and images for: Translational Control by the DEAD Box RNA Helicase belle Regulates Ecdysone-Triggered Transcriptional Cascades
Source: PLoS Genet. 2012 Nov 29;8(11):e1003085. doi: 10.1371/journal.pgen.1003085 (PMC3510042; doi:10.1371/journal.pgen.1003085)

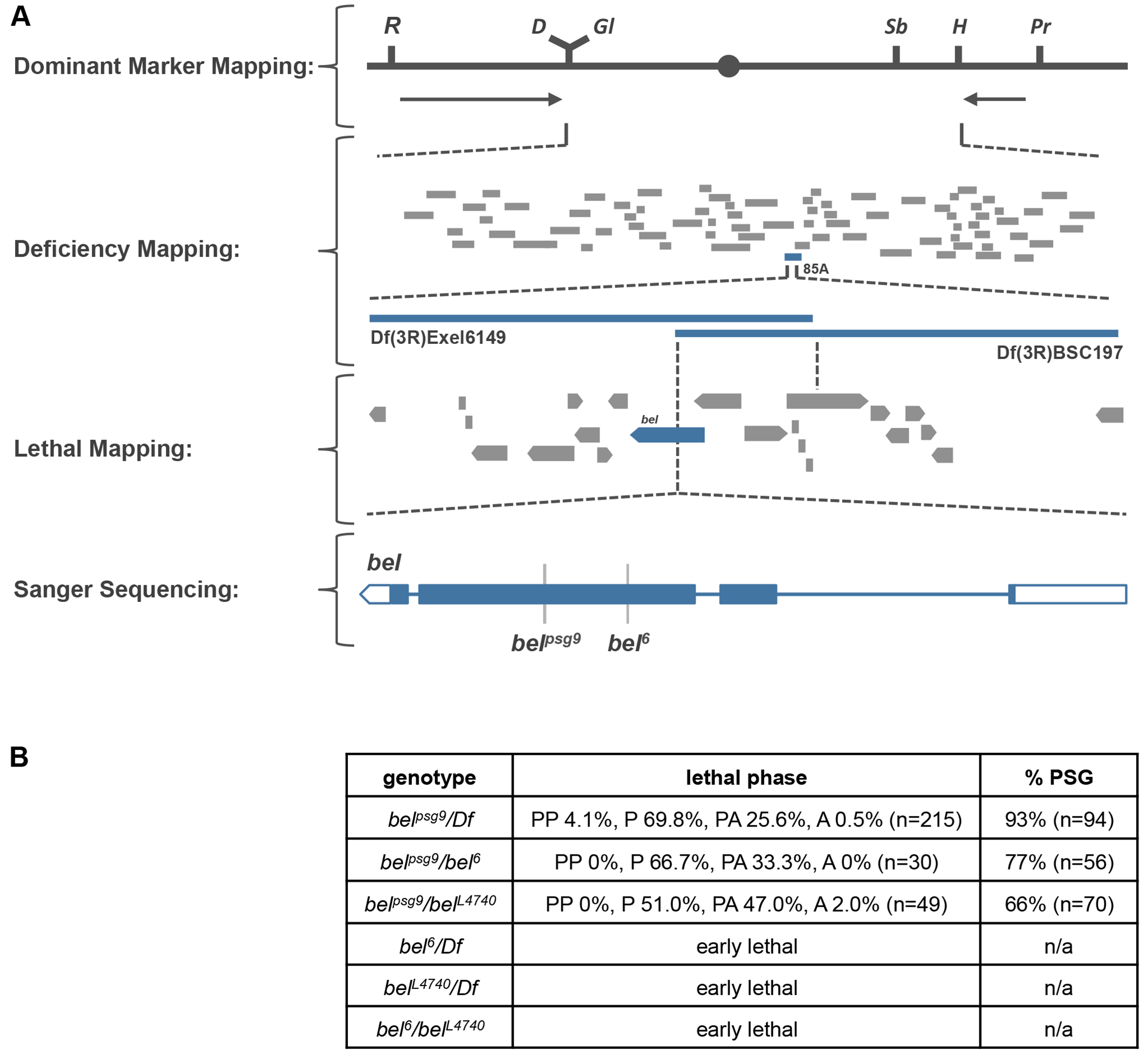

Supplement: Figure S1 — Mapping and allelic series of belpsg9. (A) psg9 mapped by recombination analysis, complementation tests and Sanger sequencing. Recombination mapping with pairs of dominant markers placed psg9 in the Glued (Gl) – Hairless (H) region of the third chromosome: right of the Roughened (R) and Dichaete (D) pair and left of the Hairless (H) and Prickly (Pr) pair (see arrows; method described in [7]). Complementation tests with chromosomal deficiencies in this region mapped psg9, first to a large cytological deficiency (Df(3R)p712), then to a small region defined by two overlapping deficiencies (Df(3R)BSC197 and Df(3R)Exel6149). All publicly available lethal mutations of genes within the minimal region were crossed and multiple alleles of the DEAD-box helicase bel failed to complement psg9. Sanger sequencing identified lesions in both belpsg9 and bel6. (B) Lethal phase and persistent salivary gland (PSG) phenotype of belle hemizygous and transheterozygous animals. All belpsg9 hemizygous and transheterozygous mutant combinations die after head eversion (as pupae) and have a highly penetrant PSG phenotype. bel6 and belL4740 hemizygous and transheterozygous animals die prior to puparium formation. PSG was assayed at 24 hours after puparium formation when control animals have 0% PSG (n = 100). Early lethal: embryonic or first larval instar lethal, PP: prepupal lethal, P: pupal lethal, PA: pharate adult lethal, A: adult escapers, n/a - not applicable because mutants do not reach the appropriate stage for PSG assay. (TIF) [file pgen.1003085.s001.tif]

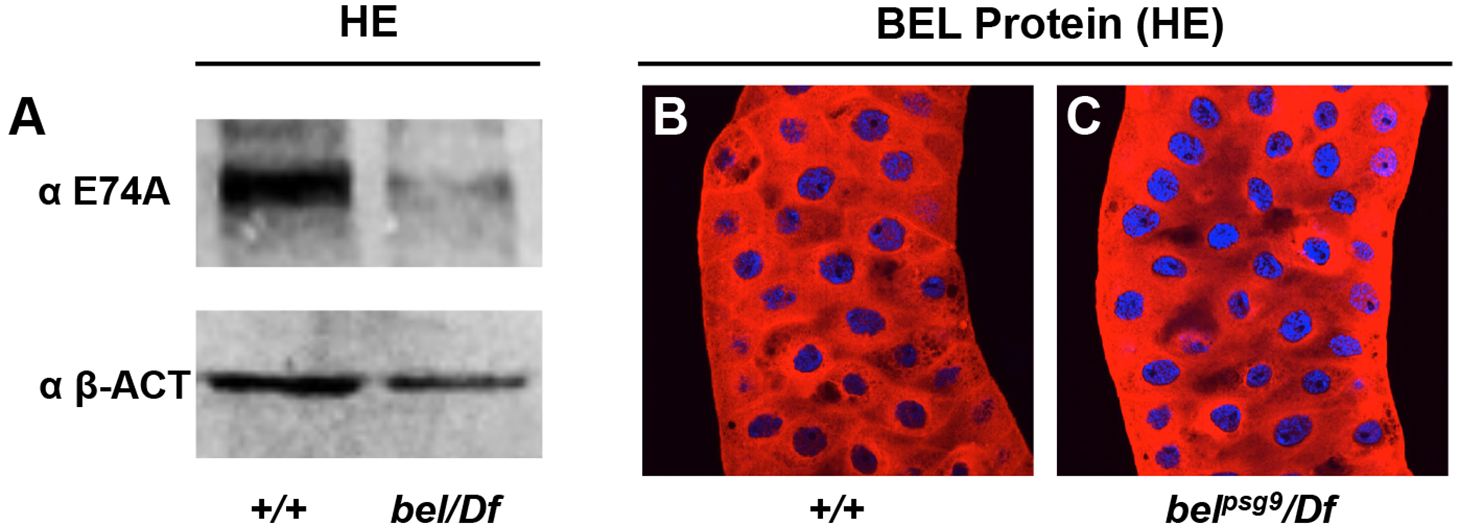

Supplement: Figure S2 — E74A and Belle protein expression in belpsg9 mutants. (A) Western blots of whole animal extracts with antibodies directed to E74A protein. E74A protein is robustly expressed in control (left) but barely detectable in belpsg9 mutant animals (right) at head eversion. β-Actin used as a loading control. (B–C) Larval salivary glands stained with antibodies directed to Belle shown in red with DAPI costained nuclei in blue. Belle protein is cytoplasmic in both control (B) and belpsg9/Df mutant (C) salivary glands at head eversion (HE). (TIF) [file pgen.1003085.s002.tif]

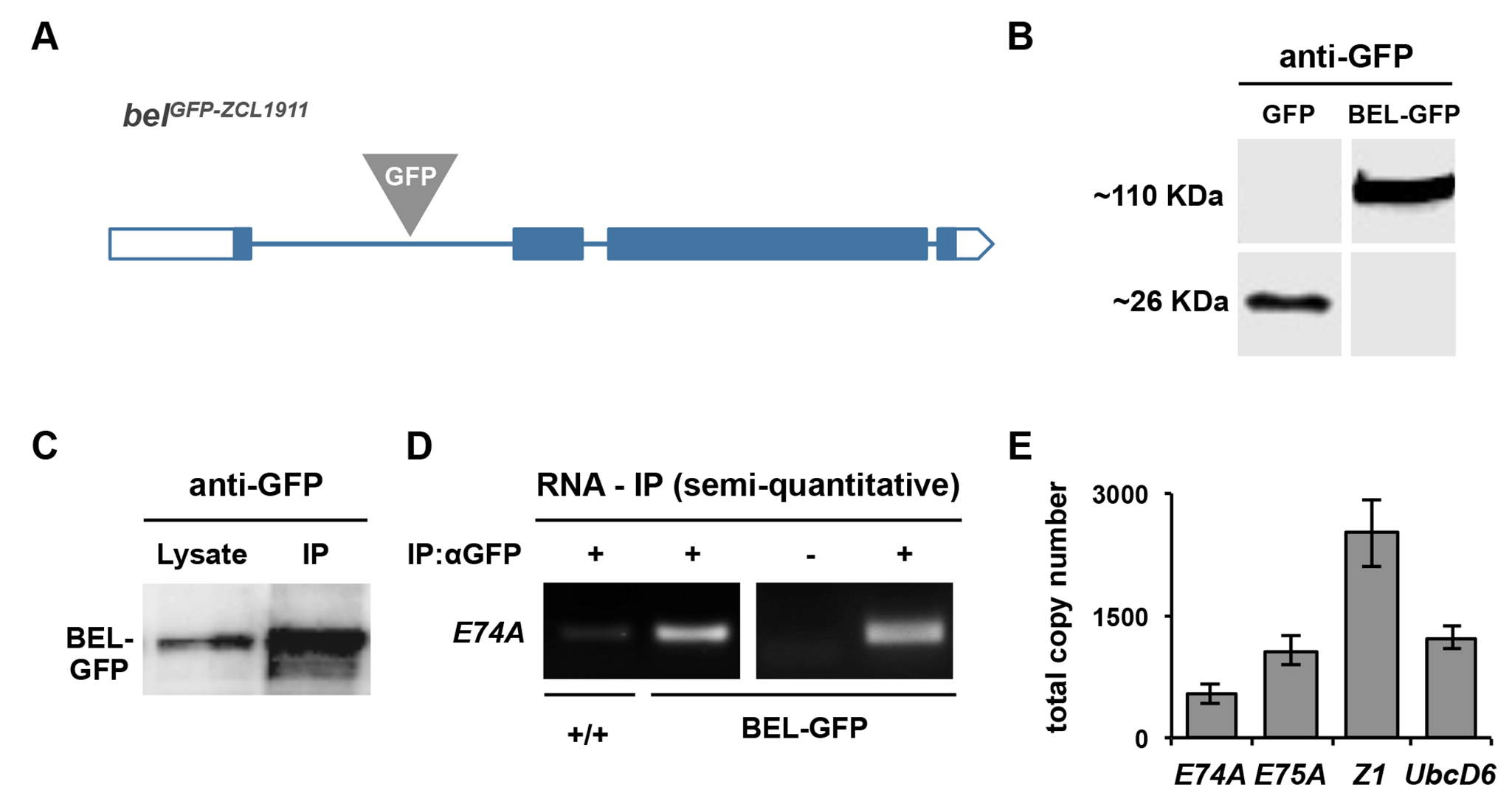

Supplement: Figure S3 — RNA binding protein immunoprecipitation experiments. (A) Schematic depicting the nature of the BEL-GFP protein trap line used (belGFP-ZCL1911) [17]. (B) Western blot analysis using antibodies directed to GFP (anti-GFP) detects a BEL-GFP fusion protein of the appropriate size in whole animal lysates (a GFP expressing line used as control). (C) Western blot analysis using anti-GFP detects BEL-GFP in whole animal lysates and in immunoprecipitated samples. As expected, immunoprecipitated BEL-GFP samples from whole animal lysates at −2 AHE enrich BEL-GFP protein. (D) Immunoprecipitated (IP) BEL-GFP containing RNPs detects enrichment of E74A transcripts using RT-PCR. Two methods were compared: extracts from control or Bel-GFP animals IP with anti-GFP antibodies (left lanes) or Bel-GFP extracts IP with and without anti-GFP antibodies (right lanes). Both approaches show strong enrichment of E74A mRNA in BEL-GFP RNPs. (E) Absolute quantification of mRNA copy number in whole animal lysates at −2 AHE. The copy number for E74A mRNA is the lowest compared to the other control genes, further supporting the enrichment in the RNA IP experiments. (TIF) [file pgen.1003085.s003.tif]

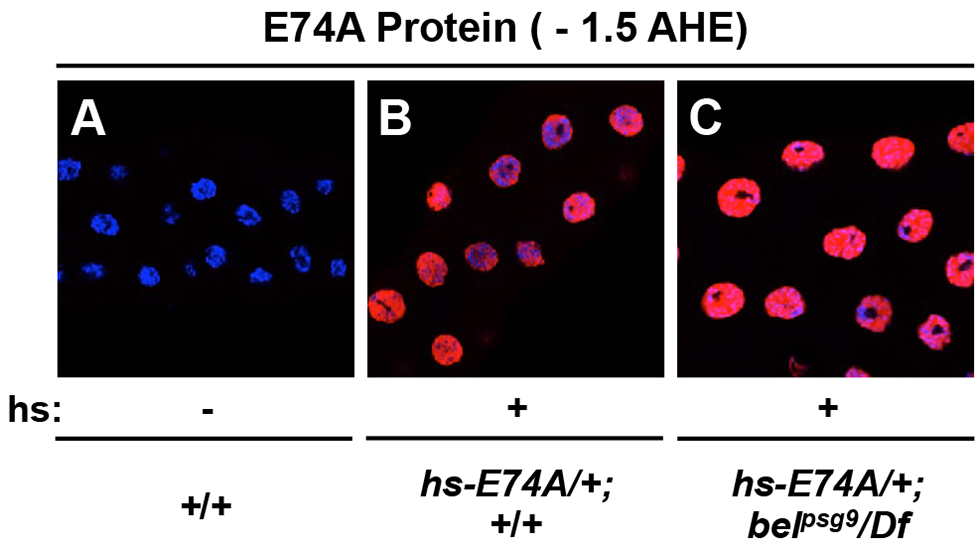

Supplement: Figure S4 — Ectopic expression of E74A protein from the hs-E74A transgene in salivary glands. Staining with antibodies directed to E74A protein shown in red demonstrates that heat-shock (hs) driven induction of the hs-E74A transgene expresses E74A protein in both control (B) and belpsg9/Df mutant (C) salivary glands at a stage (1.5 hours before head eversion) when endogenous E74A protein is not present (A). Ectopic expression paradigm as described in Figure 5A. DAPI costained nuclei in blue. (TIF) [file pgen.1003085.s004.tif]
