# Supplementary material for: Translational Control by the DEAD Box RNA Helicase belle Regulates Ecdysone-Triggered Transcriptional Cascades
Source: PLoS Genet. 2012 Nov 29;8(11):e1003085. doi: 10.1371/journal.pgen.1003085 (PMC3510042; doi:10.1371/journal.pgen.1003085)
Supplement: Table S1 — qPCR primer sequences. The first column indicates the forward (F) and reverse (R) primer pair for each target gene. Second column shows sequence for each primer. Each primer pair was designed and validated in this study unless otherwise noted. Source references: (a) [42], (b) [43], (c) [44] and (d) [45]. (DOCX) [file pgen.1003085.s005.docx]

| Gene | Primer Sequence | Source |
| --- | --- | --- |
| ark F | TGCACTTCATCGGAGTATCG | (a) |
| ark R | AGATCGATCCACTGGCATTC |  |
| BR-C F | CTCAAGAGCACACCCTGCAA | (b) |
| BR-C R | CGTGCAGGTCCATGAAGTTG |  |
| E74A F | GTTGCCGGAACATTATGGATATA | (c) |
| E74A R | GCCCTATGTCGGCTTGCT |  |
| E74B F | ATCGGCGGCCTACAAGAAG | (c) |
| E74B R | TCGATTGCTTGACAATAGGAATTTC |  |
| E75A F | AGCCGCAGCAGCAAATG | (d) |
| E75A R | ACCCGAGTGGTGCAGAT |  |
| E93 F | GCAATACAACCACAGCAGCAAG | (present study) |
| E93 R | GTGATGGTGTACTGGTGGCT |  |
| FTZ F | TGGACTACACCCTCACCTGC | (present study) |
| FTZ R | CACGTTCTCCCGGCCTCTAT |  |
| hid F | ATCCAGTCTGCCATACCGATAG | (present study) |
| hid R | AACAGTTGGCCAAGTGAAGCTC |  |
| Nc F | CTCGCTAAACGAACGGAGAAC | (present study) |
| Nc R | CAACGACACCCACATAAGGG |  |
| rp49 F | CCAGTCGGATCGATATGCTAA | (a) |
| rp49 R | ACGTTGTGCACCAGGAACTT |  |
| rpr F | ATCCGAAGACCGGAAGAAAG | (present study) |
| rpr R | GTGGCTCTGTGTCCTTGACTG |  |
| UbcD6 F | ACATATTGCAGAACCGCTGG | (present study) |
| UbcD6 R | GCTTTCACACGCTTCTCGT |  |
| Z1 F | GCCAACAACAACAGCCCAACG | (present study) |
| Z1 R | CACCGCCGCGTACATTTGCT |  |
